# Effects of Housing and Environmental Enrichment on Performance, Welfare, and Air Quality in Fattening Pigs

**DOI:** 10.3390/ani16040580

**Published:** 2026-02-12

**Authors:** Juho Lee, Huimang Song, Sarbani Biswas, Kyung-won Kang, Jinhyeon Yun

**Affiliations:** 1 Department of Animal Science, College of Agriculture and Life Sciences, Chonnam National University, Gwangju 61186, Republic of Korea; 2 Department of Agricultural and Food Sciences, University of Bologna, 40127 Bologna, Italy; 3 Animal Genetic Resources Research Center, National Institute of Animal Science, Rural Development Administration, Hamyang 50000, Republic of Korea; 4 Swine Research Center, Sunjin R&D Institute, Sunjin Co., Ltd., Icheon 17332, Republic of Korea

**Keywords:** aggressive behavior, ammonia levels, behavioral changes, enrichment materials, finishing pigs, pig welfare

## Abstract

In intensive pig production systems, limited space and lack of enrichment materials (EMs) restrict natural behaviors, inducing chronic stress and impairing welfare and health. Conventional EMs such as straw and sawdust improve comfort but increase NH_3_ and particulate emissions and hinder manure management on slatted floors. This study compared rice-straw silage (RS), sawdust (SD), and sling belt (SB) as EMs for growing-finishing pigs to evaluate their effects on growth performance, behavior, body lesions, cleanliness score of body, and pen air quality. A total of 344 crossbred pigs ([Landrace × Yorkshire] × Duroc, 30.5 ± 3.10 kg) were randomly allocated to four treatments: Control, 50% slatted and 50% solid flooring; RS, 100% solid flooring with a 7-cm layer of RS; SD, 100% solid flooring with a 7-cm layer of SD; SB, 50% slatted and 50% solid flooring with 10 SBs (1.5 m long and 75 mm wide). At week 10, the RS pigs had the lowest body weight. At week 0, the RS and SD pigs exhibited more positive behaviors, although the SD pigs also showed the highest number of injurious interactions at week 3. Between weeks 0 and 5, the SD pigs spent less time lateral lying and more time sternal lying, while during weeks 8–11, sitting was more prevalent. Both RS and SD groups exhibited lower cleanliness scores at week 6 and higher NH_3_ and CO_2_ levels at week 10. In conclusion, bedding materials such as RS and SD promoted positive behaviors during the early phase; however, prolonged use without adequate management impaired hygiene, air quality, resting behavior, and growth performance. These findings highlight the importance of the appropriate selection and management of EMs in intensive pig production systems.

## Simple Summary

Insufficient space and limited access to appropriate environmental enrichment materials (EMs) restrict the expression of natural behaviors, increase stress, and compromise the welfare of pigs in intensive production systems. In some countries, however, the practical use of conventional bedding-based EMs is constrained by difficulties in manure management and environmental control, making their long-term application challenging. This study compared different EMs to evaluate their effects on growth performance, behavior, body lesions, and pen-level air quality in growing-finishing pigs. Conventional bedding materials provided short-term benefits by stimulating positive behaviors and reducing skin lesions during the early growing period. However, these benefits were not sustained and were accompanied by negative consequences over time, including deteriorated hygiene, elevated concentrations of noxious gases, and reduced growth performance when material management was inadequate. As an alternative approach, the present study demonstrated that the use of sling belts (SBs) in pens with slatted flooring was associated with improved welfare-related outcomes and stable production performance. These findings provide practical insights into the selection and management of EMs that can be feasibly applied in intensive pig housing systems where the use of bedding materials is limited.

## 1. Introduction

Pigs housed in intensive systems have limited opportunities to express natural behaviors due to constraints in space, lack of enrichment materials (EMs), or both. Such restrictions can induce chronic stress [1,2], compromise immune function [3], and ultimately undermine both animal welfare and robustness. To address these challenges, increasing attention is being paid towards the provision of EMs that stimulate instinctive behavior and mitigate the adverse effects of confinement [4]. Reflecting these concerns, regulations in several countries, including within the European Union, require or recommend the provision of EMs to meet the pigs’ behavioral needs, with the use of bedding materials depending on the housing system [5].

Among commonly used EMs, straw and sawdust (SD) have been proven effective in reducing negative social interactions, including ear and tail biting [6,7]. These materials also enhance physical comfort by improving bedding quality, which supports natural behaviors and contributes to overall welfare [8]. However, when inadequately managed or infrequently replaced, these materials accumulate within the pens, leading to increased concentrations of NH_3_ [9], CO_2_ [10,11], and particulate matter (PM) [12], which negatively affect pig health and growth [13,14]. Bedding materials are primarily recommended for fully solid-floor systems and, in some cases, for semi-slatted floors, whereas their use in slatted-floor systems is limited due to interference with manure management and differential behavioral responses across housing types [4,15]. Accordingly, Grandin [16] has recommended using straw bedding only in farrowing pens and not gestation and finishing pens to balance welfare and waste management.

To overcome these limitations, various alternative EMs, including plastic balls, metal chains [17], hanging toys [18], hemp ropes, rubber balls [19], knotted cotton ropes [20], cloth strips [16], and belts [21], have been suggested. In a recent study, we demonstrated that sling belts (SBs), made of soft polyester mounted on pen walls reduced negative behaviors, body lesions, and physiological stress in growing pigs housed on slatted floors, thereby improving welfare and growth performance [22]. These belts allowed the pigs to manipulate them with their snouts or forelegs and rub their bodies against them while not interfering with manure management.

Intensive pig producers worldwide face challenges with the availability, cost, and management of bedding materials, which may limit their practical use despite welfare recommendations. These constraints are particularly evident in countries such as Korea, where land availability for livestock production is limited and the supply of bedding materials and post-use composting are difficult, the range of EMs that can be practically applied in intensive production systems is highly restricted. Nevertheless, Korean animal welfare standards primarily require the provision of bedding materials such as straw, without sufficiently considering the feasibility of replacement or renewal [23]. As a result, although producers provide bedding materials to comply with welfare regulations, subsequent management practices, including replenishment or replacement, are rarely implemented due to the associated economic and labor burdens. Under these conditions, it remains unclear whether the provision of conventional bedding-based EMs is effective or sustainable in intensive housing systems, highlighting the need to evaluate their practical welfare benefits and to explore feasible alternative strategies. Therefore, the present study aimed to compare the effects of rice-straw silage (RS), SD, and SB as EMs for growing-finishing pigs reared under intensive housing conditions, focusing on growth performance, behavior, body lesions, cleanliness score of body, and pen-level air quality. We hypothesized that SBs would provide behavioral stimulation comparable to RS or SD, while partially slatted flooring combined with SB would maintain lower levels of NH_3_, CO_2_, and PM, thereby improving environmental hygiene and overall pig welfare.

## 2. Materials and Methods

### 2.1. Animals, Housing, and Management

A total of 344 crossbred growing pigs ([Landrace × Yorkshire] × Duroc), comprising both female and castrated males, were included in this study. All pigs were reared in a single facility to maintain identical environmental conditions. The pigs had an initial average weight of 30.5 ± 3.10 kg and were approximately 10 weeks old at the start of the trial, which lasted 10 weeks, concluding 2 weeks before the expected slaughtering date. Tail docking and needle-teeth clipping were performed for all pigs.

**Figure 1:**
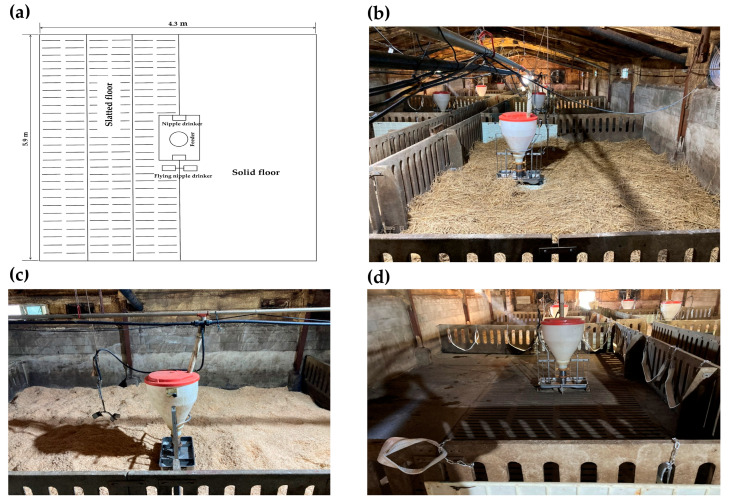
Experimental layouts for the four treatment groups: (**a**) Control, 50% slatted and 50% solid flooring; (**b**) Rice-straw silage (RS), 100% solid flooring with a 7-cm layer of RS; (**c**) Sawdust (SD), 100% solid flooring with a 7-cm layer of SD; (**d**) Sling belt (SB), 50% slatted and 50% solid flooring with 10 SBs (1.5 m long and 75 mm wide). All pens (5.9 × 4.3 m; 0.9 m^2^/pig) were equipped with a feeder, two nipple drinkers, and two suspended nipple drinkers.

The pigs were housed in a temperature-controlled facility with automated ventilation. They were randomly allocated to 12 pens, 10 of which measured 5.9 × 4.3 m, while the remaining two measured 6.9 × 4.3 m. Specifically, to maintain a constant stocking density of 0.9 m^2^ per pig, 10 pens (5.9 × 4.3 m) housed 28 pigs each, and 2 pens (6.9 × 4.3 m) housed 32 pigs each. Each pen was equipped with one feeder and two nipple drinkers in the center. Additionally, two nipple drinkers were suspended in each pen. The entire pigsty measured 9.1 × 42 m, with a ceiling height of 4 m. There were 10 windows, each measuring 1.0 × 0.4 m. The flooring of each pen was split evenly; half was slatted, and the other half was solid. A slurry system was utilized for manure management (Figure 1a,d). To provide RS or SD as EMs, the slatted floor was temporarily blocked using plastic panels, measuring 1800 × 600 mm (Figure 1b,c) to prevent drainage, effectively creating a solid floor environment where moisture was managed solely by the absorption capacity of the bedding materials. All pigs had *ad libitum* access to feed and water. Feed was manually carried into the feeder by a researcher to measure feed intake.

### 2.2. Experimental Design and Treatment Management

The growing pigs were randomly assigned to one of the following four treatment groups using a randomized complete block design (RCBD) with three blocks based on pen location (entrance, middle, and end sections) to ensure uniform environmental conditions (3 replicate pens per group): (1) Control (*n* = 88, housed in pens having 50% slatted and 50% solid flooring); (2) RS (*n* = 84, housed in pens having 100% solid flooring with a 7-cm layer of RS [moisture content approx. 60–70%]); (3) SD (*n* = 84, housed in pens having 100% solid flooring with a 7-cm layer of SD); and (4) SB (*n* = 88, housed in pens having 50% slatted and 50% solid flooring and provided with 10 SBs, 1.5 m long, and 75 mm wide).

The SB (Youngil Industry Co., Yeongcheon, Republic of Korea), made of 100% polyester, was mounted on the side walls at a height of 20 cm above the floor using metal chains. This setup facilitated pig interaction with the belts while minimizing contamination. RS was replenished every 10 d due to its rapid decrease in volume, while SD was not periodically replenished. However, 7 weeks after the trial started, both EMs were replaced entirely because the pigs could no longer assume a comfortable resting position, and their faces would come into contact with manure when they lay on their sides.

### 2.3. Data Collection

#### 2.3.1. Body Weight and Feed Intake

Body weight and feed intake were measured at weeks 0, 3, 6, and 10 of the experiment. The pigs were individually weighed using a scale (CAS Co., Yangjoo, Republic of Korea; minimum division: 0.1 kg), and feed intake was determined by calculating the difference between total feed supplied and remaining feed in the feeder. Feed intake data were assessed at the pen level. From these data, average daily gain (ADG), average daily feed intake (ADFI), and gain-to-feed ratio (G:F) were calculated.

#### 2.3.2. Posture and Behavior

The pigs were video recorded continuously for 24 h (from 08:00 on the first day to 08:00 the next day) at weeks 0, 3, 5, 8, and 11 using an IPTV cloud storage camera (HN0-E60; Hanwha Techwin, Seongnam, Republic of Korea). The posture data were categorized into the early fattening period (weeks 0–5), late fattening period (weeks 8–11), and overall period (weeks 0–11). Pig posture and behavior were analyzed by reviewing videos during the first 10 min of every hour over the 24-h period following the Welfare Quality^®^ (2009) assessment system [24]. To accurately quantify the data, two distinct observation methods were applied at 2-min intervals: Posture was recorded using instantaneous scan sampling (checking the frame at the specific time point) to estimate the proportion of time. Behaviors were recorded using continuous observation of 5-s video clips. The camera’s display resolution was 1920 × 1080 pixels, and the frame speed was 25 fps. Posture and behavior data were assessed at the pen level. Detailed criteria for behavioral observations are presented in Table 1.

#### 2.3.3. Body and Ear Lesions

Body and ear lesions were evaluated when the pigs were on the scale for weight measurement. Lesions were categorized into the following five areas based on their location: ear, front (head to neck), middle (behind neck to in front of hip), rear (hip to tail), and legs. Lesion severity was scored based on the weighted number of lesions according to the Welfare Quality^®^ (2009) criteria [24]. The scoring system is described below:

Body lesion scores:

- Score 0: 0–4 lesions;
- Score 1: 5–10 lesions;
- Score 2: More than 11 lesions.

Ear lesion scores:

- Score 0: No lesions;
- Score 1: 1 or more ear lesions;
- Score 2: 1 or more bleeding ear lesions.

For further clarification, a score of ‘1 lesion’ referred to small or minor wounds (e.g., a single scratch > 2 cm, two parallel scratches ≤ 0.5 cm apart, or a lesion < 2 cm). A score of ‘5 lesions’ reflected moderate injury severity, including a total of five lesions, a bleeding wound measuring 2–5 cm, or a healed wound larger than 5 cm. Specifically, larger or deeper wounds were counted as multiple lesions (e.g., a deep wound > 5 cm counted as 16 lesions) to incorporate severity into the total count. Lesions were assessed at the individual level.

#### 2.3.4. Cleanliness Score of Body

The cleanliness of the pigs was measured at weeks 0, 3, 6, and 10 by a trained observer. The pig’s body was divided into the following four zones: legs, left side, right side, and head. Each zone was inspected to determine whether more than 50% of the area was stained with manure. Cleanliness was scored from 0 (all zones dirty) to 4 (no dirty zones) based on the number of body zones stained with manure. Cleanliness score data were assessed at the individual level.

#### 2.3.5. Pen Air Quality at Week 10

At week 10, air quality was evaluated at 09:00 and 15:00 in each pen. In the RS and SD groups, measurements were taken at three representative zones per pen, with two readings performed per zone (total 6 readings per pen). In the SB and control groups, measurements were taken at six zones (three on the solid floor and three on the slatted floor) with two readings per zone (total 12 readings per pen). All readings within a pen were averaged to provide a single representative value per replicate for statistical analysis.

The concentrations of NH_3_, CO_2_, and PMs (PM_2.5_ and PM_10_) as well as temperature and relative humidity were measured and recorded. Measurements were taken using a gas detection instrument (K-600, Bosean Electronic Technology Co., Ltd., Zhengzhou, China) with a resolution range of 0–100 ppm for NH_3_ and 0–5000 ppm for CO_2_, both with an accuracy of ±5%. PM concentrations were measured using an optical detection device (HT-9600, Dongguan Xintai Instrument Co., Ltd., Dongguan City, China) with a resolution range of 0–1000 mg/m^3^ and an accuracy of ±12%. The device measures temperature within a range of 0–50 °C and relative humidity from 0–100%, with accuracies of ±0.5 °C and ±3%, respectively.

### 2.4. Statistical Analysis

All of the data were analyzed using JMP Pro, version 17 (SAS Institute Inc., Cary, NC, USA). The study followed a randomized complete block design with three pens per treatment group. Treatment was considered as a fixed effect, and a pen, a random effect. The block effect was excluded from the final statistical model to maintain model parsimony. The PROC MIXED model was applied to analyze body weight, ADG, ADFI, G:F, posture proportion (lateral, sternal, sitting, and standing/walking), behavior proportions (injurious interaction, positive interaction, exploration, eating/drinking, and inactivity), skin lesion scores, and environmental conditions (NH_3_, CO_2_, PM_2.5_, PM_10_, humidity, and temperature). For posture analysis, a repeated measure analysis with a compound symmetry structure model was used to analyze the data of weeks 0–5, 8–11, and 0–11. The cleanliness of pigs was analyzed using the Kruskal–Wallis test, incorporating the weighted mean values. Data were presented as the means and standard errors of the mean (SEM).

## 3. Results

### 3.1. Growth Performance

The EMs had no significant effect on average body weight, ADG, ADFI, and G:F at weeks 0, 3, and 6 (Table 2). However, by week 10, the RS group tended to have lower average body weights compared to the other treatment groups (*p* < 0.10, Table 2). Over the entire experimental period (weeks 0–10), no significant differences were observed among treatments for ADG, ADFI, or G:F (Table 2).

### 3.2. Posture and Behavior

During weeks 0–5, pigs in the SD group spent a smaller percentage of time in the lateral lying posture than the control and SB groups (*p* < 0.001), while the time spent in the sternal lying posture was the longest in SD (*p* < 0.001; Table 3). Furthermore, the SD group pigs spent a higher percentage of time sitting than the SB group pigs during both weeks 8–11 and the overall period (*p* < 0.05 for both; Table 3). No significant differences were observed in standing/walking postures across the groups during the study (Table 3).

At week 0, pigs in the RS and SD groups showed a higher proportion of positive interaction (*p* < 0.05 for both) and exploration behavior (*p* < 0.01 for both) than those in the control group (Table 4). Furthermore, pigs in the RS group showed a higher percentage of eating/drinking behavior than those in the control and SB groups at week 0 (*p* < 0.001; Table 4). Conversely, pigs in the RS and SD groups exhibited a significantly lower percentage of inactivity than those in the control and SB groups at week 0 (*p* < 0.001 for both; Table 4).

At week 3, pigs in the SD group showed the highest percentage of injurious interaction (*p* < 0.05), while those in the RS group showed the highest proportion of exploration behavior compared with the other groups (*p* < 0.05; Table 4). Inactivity levels were lower in the RS group than in the control and SB groups at week 3 (*p* < 0.05; Table 4).

At week 5, pigs in the RS group continued to display a higher proportion of exploration behavior than those in the control and SB groups (*p* < 0.001; Table 4). Compared with the control group, the RS and SD groups demonstrated lower inactivity at week 5 (*p* < 0.05 for both; Table 4).

The trend in week 8 was similar to that in week 5, with pigs in the RS and SD groups showing a tendency for increased exploration than those in the control group (*p* < 0.10 for both) and the SB group also exhibiting an increased tendency to decrease eating/drinking behavior compared with the other treatment groups (*p* < 0.10; Table 4). Pigs in the RS group were more active than those in the SB group at week 8, as evidenced by the significantly lower inactivity level (*p* < 0.05; Table 4). At week 11, the proportion of exploration behavior was higher in the SD group than that in the control group (*p* < 0.05; Table 4).

### 3.3. Body and Ear Lesions

At week 3, pigs in the RS and SD groups tended to have a lower proportion of body lesion score 0 than those in the control and SB groups (*p* < 0.10 for both; Table 5). At week 10, however, pigs in the RS group had a higher proportion of body lesion score 0 than those in the control group (*p* < 0.05), while the proportion of pigs with body lesion scores 1 and 2 were lower in the RS group than those in the control group (*p* < 0.05; Table 5). During the experimental period (weeks 0–10), the proportion of pigs with body lesion score 0 tended to be higher in the RS and SB groups than those in the control and SD groups (*p* < 0.10 for both; Table 5). Furthermore, pigs in the SB group had a lower proportion of body lesion score 1 than those in the control group (*p* < 0.05; Table 5).

There were no significant differences in the proportions of pigs with ear lesion scores 0, 1, and 2 for the entire experimental period (Supplementary Table S1).

### 3.4. Cleanliness Score of Body

**Figure 2:**
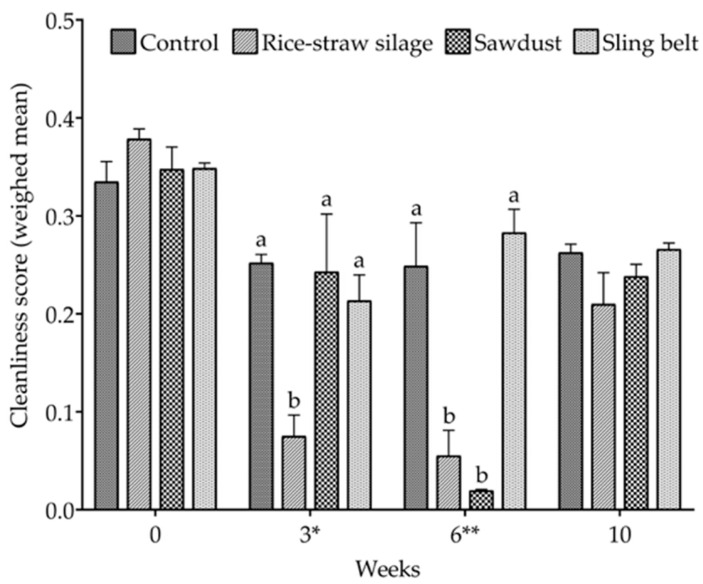
Cleanliness scores (weighted means) of fattening pigs housed in pens with different enrichment materials (experimental groups: control, rice-straw silage, sawdust, and sling belt) measured at 0, 3, 6, and 10 weeks. Different superscript letters (a, b) indicate significant differences among treatments within the same time point (*p* < 0.05). Values are presented as the means ± standard error of the mean (SEM). * *p <* 0.10, ** *p <* 0.05. High numbers = clean animals.

There was no significant difference in the cleanliness of pigs at 0 and 10 weeks between the treatment groups (Figure 2). However, the weighted mean cleanliness score tended to be the lowest in the RS group compared with that in the other treatment groups at week 3 (*p <* 0.10, Figure 2). The RS and SD group had significantly lower cleanliness scores at 6 weeks compared with the control and SB groups (*p <* 0.05 for both, Figure 2).

### 3.5. Pen Air Quality at Week 10

At week 10, NH_3_ concentrations measured at 09:00 tended to be higher in the RS and SD groups than those in the control and SB groups (*p* < 0.10 for both; Table 6). No other significant differences were observed at 09:00.

At 15:00, NH_3_ levels in the RS and SD groups were higher than those in the SB group at week 10 (*p* < 0.01 for both; Table 6). CO_2_ levels in the RS and SD groups were also higher than those in the control group at 15:00 (*p* < 0.05 for both; Table 6). Furthermore, the relative humidity in the RS and SD groups tended to be higher than that in the control and SB groups at 15:00 (*p* < 0.10 for both; Table 6). The PM_2.5_ levels in the control and SB groups tended to be higher than those in the RS and SD groups (*p* < 0.10 for both), and the PM_10_ levels in the control and SB groups were significantly higher than those in the RS and SD groups at 15:00 (*p* < 0.05 for both; Table 6).

## 4. Discussion

### 4.1. Growth Performance

Enriched environments in modern pig farms are known to reduce negative social behaviors in pigs and fulfil behavioral needs, thereby lowering stress levels that can impact welfare and growth performance [17,25]. Similarly, pigs raised in pens with bedded floor systems using straw and SD showed a higher daily weight gain and feed intake along with increased exploratory and manipulative behaviors compared with those housed in pens with slatted-floor systems [4,26]. However, in the present study, pigs in the RS group tended to have lower BW at 20 weeks of age, despite showing increased exploratory behavior during certain weeks compared with the other groups. One possible explanation for this outcome may be the influence of bedding materials on the microenvironment in the pens. Previous studies have shown that straw-based floor systems generally have approximately 30% higher NH_3_ concentrations than slatted systems due to increased aerobic microbial activity within the litter [10,27]. Elevated NH_3_ levels can irritate pigs’ respiratory tracts and mucous membranes, potentially leading to reduced feed intake [28]. Even if the intake is not reduced, high NH_3_ levels in barns may still impair feeding efficiency by inducing respiratory tract damage, oxidative stress, and inflammation, thereby increasing metabolic energy expenditure and hindering growth [29,30].

Furthermore, during hotter seasons, bedding materials, such as straw, may exacerbate heat stress by acting as thermal insulation, thereby raising the pen temperatures [31]. This consideration is particularly relevant because the present trial was conducted during the hottest period of the year. In contrast, concrete floors can facilitate heat dissipation due to their lower surface temperatures [32], which may partly explain the better growth performance in pigs housed without bedding. A similar situation may occur with SD, where increased NH_3_ concentrations and its potential insulating properties could influence the housing environment; however, no change in BW was observed in this study. In the SB group, lower NH_3_ levels were observed under the combined application of SB and a partly slatted flooring system, with no associated change in BW. These findings indicate the need for future studies to identify the most effective EMs under different housing and seasonal conditions for improving both welfare and productivity.

### 4.2. Posture and Behavior

Pigs housed in pens with SD flooring were observed to sit more frequently and displayed less lateral lying but more sternal lying compared with those in the SB group. These behavioral trends conform with the findings of Wei et al. [33], who reported similar patterns in their study involving pigs reared in pens with deep-litter versus slatted-floor systems without EMs. Lateral lying represents a state of full relaxation and immobility, and sternal lying describes a posture in sows where they stay awake, alert, and responsive to their environment, such as engaging with EMs through activities like chewing [34]. Moreover, pigs generally adopt a sitting posture when experiencing physical discomfort or psychological anxiety [35]. Thus, the current findings suggest that inadequate management of the SD-based floor might have hindered the pigs’ ability to rest comfortably.

In this study, SD was spread on the floor at a shallow depth of 7 cm. Consequently, when the SD was mixed with manure, it fermented and attained a sludge-like consistency before it could dry. This mixture of sludge and SD accumulated in the pen until it was removed and replaced with fresh SD. When pigs lie laterally in this environment, their eyes, nose, and mouth can become submerged in the sludge, which can likely make breathing difficult. This may explain their preference for sitting and sternal lying postures. Previous research has demonstrated that the proper management of bedding, through frequent cleaning and replenishment, not only enhances lying comfort but also encourages pigs to adopt more comfortable resting postures, thereby improving their welfare status [36,37]. However, this approach may increase labor demands and overall resource input requirements, posing challenges for large-scale or resource-limited farms.

These differences were not observed when comparing the SB and control groups, suggesting that the apparent advantage of SB over SD may not be solely attributable to its structural characteristics or position in the pen but rather the slatted floor of the pen, which was also present in the control group. Slatted flooring has been associated with improved hygienic conditions, reduced moisture accumulation in the lying area, and enhanced lying comfort, thereby facilitating the adoption of more relaxed and sustained resting postures in pigs [15,21]. No significant postural changes were observed in the RS group, likely due to insufficient straw depth, moisture accumulation, and reduced lying comfort associated with thermal insulation effects. These findings suggest that without proper management, RS may not produce noticeable improvements in resting postures compared with slatted floors. Future studies should therefore evaluate whether increasing bedding depth or optimizing replacement frequency can improve comfort and hygiene while reducing negative environmental effects.

In this study, pigs housed in pens with floors covered with bedding materials (RS and SD) were found to have increased positive interaction and exploratory behavior in the first week compared with the other groups, which aligns with the findings of Morrison et al. [38], who reported that the provision of SD promotes positive behaviors in pigs. Similarly, Casal-Plana et al. [19] observed that pigs reared with EMs, such as SD, exhibited fewer stereotypic and redirected behaviors, along with increased exploratory activity. Consistent with the findings of Machado et al. [18], pigs in the RS and SD groups in the current study exhibited higher eating and drinking behaviors and lower inactivity at week 0, which were positively correlated with exploration behavior. Exploration, which is the instinctive behavior of pigs, can reduce stress by providing them with the satisfaction of a sense of accomplishment [39]. The reduction of stress in pigs has been reported to increase their feed intake [25]. However, increased exploratory activity often requires higher energy expenditure, which could partially explain the absence of an effect on growth performance despite the positive welfare effects observed in the present study. Meanwhile, pigs in the SB group exhibited reduced eating/drinking behavior, which was accompanied by increased inactivity.

In the current study, pigs in the SD group showed the highest level of injurious interactions at week 3, which coincided with increased sitting and sternal resting postures, suggesting discomfort or restlessness. By this time, the SD bedding had become heavily soiled with manure, likely reducing lying comfort and contributing to increased injurious interactions. Nevertheless, as the trial progressed, the pigs in pens with RS flooring exhibited increased exploratory activity and a gradual reduction in inactivity. Meanwhile, the SD group followed a similar pattern overall, except for a notable shift at week 3 when exploration decreased and injurious interaction increased prior to bedding replacement at week 7. Although SD and RS were refreshed at that point to maintain hygiene and functionality, the deterioration observed in the earlier weeks likely influenced pig comfort and behavior, and the replacement might have moderated these effects in the later phase of the trial. In contrast, pigs in the SB and control groups showed similar levels of activity and exploration. However, pigs in the SB group exhibited a higher proportion of lateral lying. This difference may be attributed to the slatted flooring found in both groups, which offers a cleaner resting area.

### 4.3. Body and Ear Lesions

Although body lesions are commonly regarded as direct indicators of agonistic behaviors (e.g., fighting or biting), they can also result from increased activity in pigs, even in the absence of aggressive interactions [40,41]. In the present study, pigs in the RS and SD groups tended to have reduced body lesions by week 3 compared with those in other groups, with the RS group demonstrating a further reduction in lesion scores by week 10. This reduction may be attributed to the prolonged engagement in straw-rooting behavior, which has been shown to mitigate redirected behaviors, such as ear and tail biting [42]. The current findings align with those of Fàbrega et al. [43], confirming that straw provision can reduce both skin lesions and redirected behaviors by enhancing opportunities for exploratory activity. Furthermore, pigs reared under enriched conditions have consistently demonstrated a decrease in redirected behaviors towards pen mates, including tail biting, ear chewing, and belly-nosing [1,44,45].

Notably, over the entire trial period (weeks 0–10), the SB group demonstrated the most consistent and significant reduction in body lesions. Unlike minimal EMs, such as paper or chains, which offer limited manipulation opportunities and are linked to a higher incidence of lesions [43], SBs are durable and resist rapid deterioration, ensuring pigs can continually interact with them. Since they are suspended, they can be kept cleaner and are more accessible. Moreover, the movement provided by hanging SBs stimulates curiosity and manipulation. Hence, they hold pigs’ interest over time, reducing stress and the likelihood of redirected behaviors, such as injurious interactions.

### 4.4. Cleanliness Score of Body

Poor hygiene and environmental conditions can compromise the mental and physical health of pigs, with cleanliness playing a crucial role in mitigating stress, reducing disease risk, and preventing behavioral disorders [46]. Prolonged exposure to poor hygiene conditions, especially through fecal–oral contamination, has been associated with enteric diseases, liver lesions caused by ascarid infections, and decreased growth performance [26,47]. In the present study, pigs housed in pens with RS or SD bedding exhibited poorer body cleanliness at weeks 3 and 6 than those in pens with slatted floors, including the control and SB groups. This finding aligns with the observations of van de Weerd et al. [48], who noted increased soiling in the front and middle parts of straw-bedded pens, which was likely because pigs avoided dunging in the slatted rear area, disrupting the natural separation of dunging and resting zones. While the use of litter or straw as rooting material can enhance cleanliness when appropriately managed, poor control over dunging areas can have the opposite effect [49].

Pigs instinctively separate dunging, resting, and feeding areas [50]; however, this behavior can be disrupted in bedding-based systems where spatial boundaries are unclear, leading to increased soiling and reduced welfare [51]. Pig cleanliness is more strongly associated with the amount of solid floor space per pig than the extent of slatted flooring. AS, adequate space allowance, plays an important role in maintaining pig welfare and health [52]. It is also influenced by structural and environmental conditions, such as bedding material, pen layout, air velocity, and temperature [49]. Accordingly, the relatively higher cleanliness levels observed in the SB group may be related to the slatted-floor design, which facilitates the separation of functional areas and prevents the accumulation of moisture-retaining organic bedding. By avoiding fermentable materials, such as straw or SD, this system likely contributed to improved drainage and reduced soiling compared with RS and SD. These findings suggest that slatted-floor housing with SB provision can help maintain cleaner pen conditions, although the effect may be more strongly attributable to flooring type than the EM itself.

### 4.5. Pen Air Quality at Week 10

Pigs in intensive housing are often exposed to airborne pollutants, including PMs and gases such as NH_3_ and CO_2_, which impair health, welfare, and performance [14]. In this study, NH_3_ and CO_2_ concentrations were highest in the RS and SD groups, where manure accumulation on the floor was greatest. Although these gas concentrations exceeded the recommended levels reported by Buoio et al. [53] (NH_3_: 10–20 ppm; CO_2_: <3000 ppm), this may be attributable to the sampling location (10 cm above the floor). Nevertheless, the observed values remain within the upper range of concentrations previously reported in commercial pig buildings [54]. NH_3_ arises from the enzymatic hydrolysis of urea by fecal urease, while CO_2_ results from microbial decomposition [55,56]. Poor manure management in bedding systems can intensify these processes through aerobic microbial activity [9,11,57]. Accordingly, environmental measurements collected during the final week (week 10) indicated poorer pen air quality in the RS and SD groups. However, because air quality was assessed at a single time point, it was not possible to evaluate temporal changes or determine whether the observed differences were attributable to enrichment type alone, independent of flooring system and manure management. Replacement intervals for RS and SD varied with material type and contamination level, and all the pigs from the different treatment groups were housed in the same building with effective ventilation. Therefore, the environmental conditions for each pen were therefore assessed during the final week of the trial (week 10). Although this represents a limitation in terms of temporal scope, this approach enabled a comparative assessment of worst-case conditions across treatments.

Uneven dung distribution in the straw-bedded pens likely contributed to elevated NH_3_ levels. These pollutants can cause respiratory tract damage, reduce lung function, and weaken immunity, thereby impairing pig welfare and growth performance [14,28,58]. This may explain the present findings that pigs in the RS group tended to have the lowest BW at 20 weeks of age. Seasonal temperature conditions may further restrict the use of large amounts of straw because this can heighten the risk of heat stress [43]. Similarly, in our study, humidity tended to be higher in the RS and SD groups, which might have compounded thermal discomfort in the pigs.

In contrast to toxic gas concentrations, PM_10_ levels were highest in the groups having pens with slatted floors (control and SB), likely due to differences in relative humidity, which influences the suspension and settling of airborne particles. At higher humidity, water vapor binds to PM, increasing particle weight and accelerating deposition, thereby reducing airborne concentrations [11]. This mechanism may explain the relatively lower PM concentrations observed in the bedding groups, where relative humidity tended to be higher. Nonetheless, this relationship is complex and may vary with ventilation rate and environmental temperature, highlighting the need for more controlled research to clarify these interactions. A key limitation of the present study is that environmental measurements were collected only once during the peak period of bedding use. Future studies should therefore incorporate repeated assessments across different growth stages and seasons.

## 5. Conclusions

The present study demonstrates that growing-finishing pigs exhibit distinct behavioral, hygienic, and performance responses under different enrichment and housing conditions, underscoring the importance of selecting enrichment strategies that achieve a practical balance between behavioral needs, environmental hygiene, air quality, and productivity. Bedding-based EMs, such as RS and SD, induced short-term improvements in positive behaviors and reductions in skin lesions during the early growing period. However, when the replenishment or replacement of these materials was insufficient, their prolonged use was associated with progressive floor contamination, reduced cleanliness scores, deterioration of pen-level air quality measured at week 10, and impaired growth performance. In contrast, the provision of SB on partially slatted flooring was associated with consistently favorable welfare-related outcomes, including reduced physical injuries, cleaner body conditions, and favorable pen-level air quality at week 10, without compromising growth performance. Nevertheless, these advantages should not be interpreted as the effect of SBs alone, as partially slatted flooring likely played a substantial role in facilitating manure removal and maintaining hygienic pen conditions. Future studies should focus on elucidating the long-term behavioral and physiological consequences of SB-based enrichment across seasons and on assessing its potential integration with other EMs under diverse commercial conditions. In addition, experimental designs that independently assess bedding-based and non-bedding enrichment strategies across seasons and housing systems are required to clarify their long-term effects on welfare and productivity.

## Figures and Tables

**Table 1:** Behavioral ethogram of fattening pigs based on the Welfare Quality^®^ assessment system (2009).

Posture/Behavior	Ethogram
Lateral lying	With all four legs visible, lying on either side
Sternal lying	Lying on the stomach with front legs folded under the body
Siting	Partly erect on stretched front legs with the caudal end of the body contacting the floor
Standing/walking	Maintaining an upright body position with claws contacting the floor only/moving using four legs.
Injurious interaction	Aggression, including biting and disturbing other pigs
Positive social interaction	Sniffing, nosing, licking, and moving gently away from the pig
Exploration	Play and investigation, including sniffing, nosing, licking, or chewing the pen and exploring the enrichment material
Eating/Drinking	Head in the feeding and drinking trough and touching the nipple on the suspended nipple drinker
Others (inactivity)	Behaviors not related to the specified activities, typically characterized by minimal physical or exploratory actions

**Table 2:** Effects of the provision of EMs on growth performance in fattening pigs are displayed by treatment and experimental week.

Item	Control	RS	SD	SB	SEM	*p* Value
	*n* = 88	*n* = 84	*n* = 84	*n* = 88		
Initial BW, kg	30.5	30.5	30.2	30.9	0.53	0.676
Week 3						
BW, kg	46.2	45.9	46.3	46.4	0.95	0.863
ADG (wk 0–3), kg	0.746	0.731	0.766	0.744	0.02	0.686
ADFI (wk 0–3), kg	1.526	1.514	1.558	1.520	0.03	0.750
G:F (wk 0–3), %	48.8	48.2	49.0	48.9	1.00	0.911
Week 6						
BW, kg	63.0	62.2	62.1	62.7	0.92	0.284
ADG (wk 3–6), kg	0.841	0.815	0.788	0.862	0.03	0.430
ADFI (wk 3–6), kg	1.891	1.931	1.929	1.969	0.05	0.823
G:F (wk 3–6), %	44.4	42.2	40.9	43.8	1.69	0.379
Week 10						
BW, kg	82.1	80.6	82.3	82.1	1.17	0.082
ADG (wk 6–10), kg	0.661	0.636	0.698	0.687	0.03	0.698
ADFI (wk 6–10), kg	1.816	1.820	1.919	1.823	0.06	0.710
G:F (wk 6–10), %	36.4	34.8	36.3	37.6	1.05	0.462
Overall						
ADG (wk 0–10), kg	0.738	0.716	0.744	0.754	0.01	0.344
ADFI (wk 0–10), kg	1.750	1.760	1.814	1.774	0.03	0.753
G:F (wk 0–10), %	42.1	40.7	41.0	42.5	0.53	0.121

EMs, enrichment materials; RS, rice-straw silage; SD, sawdust; SB, sling belt, SEM, standard error of the mean; BW, body weight; ADG, average daily gain; ADFI, average daily feed intake; G:F, Gain-to-Feed ratio; wk, week. *n* refers to the total number of animals assessed. The statistical model included ‘Pen’ as a random effect, ensuring that the pen served as the valid experimental unit.

**Table 3:** Effects of the provision of EMs on posture in fattening pigs are displayed by treatment and experimental week.

Item	Control	RS	SD	SB	SEM	*p* Value
	*n* = 88	*n* = 84	*n* = 84	*n* = 88		
Weeks 0–5, %						
Lateral lying	63.2 ^a^	59.2 ^ab^	53.4 ^b^	61.1 ^a^	1.25	<0.001
Sternal lying	23.0 ^b^	24.3 ^b^	29.7 ^a^	24.4 ^b^	1.02	<0.001
Sitting	2.0	2.2	2.6	1.7	0.22	0.108
Standing/walking	11.6	14.1	14.2	12.7	0.55	0.287
Weeks 8–11, %						
Lateral lying	58.4	64.0	65.5	64.5	2.20	0.159
Sternal	30.7	25.1	24.0	25.8	1.51	0.142
Sitting	1.3 ^ab^	1.2 ^ab^	1.7 ^a^	0.8 ^b^	0.22	0.022
Standing/walking	9.5	9.5	8.7	8.8	1.02	0.847
Weeks 0–11, %						
Lateral lying	61.3	61.1	58.2	62.5	1.05	0.293
Sternal lying	26.1	24.6	27.4	24.9	0.61	0.327
Sitting	1.7 ^ab^	1.8 ^ab^	2.2 ^a^	1.3 ^b^	0.19	0.030
Standing/walking	10.7	12.3	12.0	11.1	0.62	0.609

^a, b^ Within a row, values with different superscript letters differ (*p* < 0.05). Data are presented as mean  ±  SEM. EMs, enrichment materials; RS, rice-straw silage; SD, sawdust; SB, sling belt; SEM, standard error of the mean. *n* refers to the total number of animals assessed. The statistical model included ‘Pen’ as a random effect, ensuring that the pen served as the valid experimental unit.

**Table 4:** Effects of the provision of EMs on behavior in fattening pigs are displayed by treatment and experimental week.

Item	Control	RS	SD	SB	SEM	*p* Value
	*n* = 88	*n* = 84	*n* = 84	*n* = 88		
Week 0, %						
Injurious interaction	0.3	0.3	0.3	0.2	0.05	0.419
Positive interaction	0.4 ^b^	0.7 ^a^	0.8 ^a^	0.6 ^ab^	0.09	0.023
Exploration	7.0 ^c^	13.5 ^ab^	14.0 ^a^	9.4 ^bc^	1.01	<0.010
Eating/drinking	6.7 ^b^	9.6 ^a^	8.0 ^ab^	5.9 ^b^	0.39	<0.001
Inactivity	85.3 ^a^	75.6 ^b^	76.7 ^b^	83.6 ^a^	1.13	<0.001
Week 3, %						
Injurious interaction	0.1 ^b^	0.3 ^b^	1.0 ^a^	0.4 ^b^	0.08	<0.050
Positive interaction	2.0	2.0	2.0	1.6	0.35	0.790
Exploration	4.1 ^b^	9.6 ^a^	5.6 ^b^	4.5 ^b^	0.72	<0.050
Eating/drinking	5.2	4.6	6.0	5.3	0.30	0.102
Inactivity	88.3 ^a^	83.3 ^b^	85.1 ^ab^	87.9 ^a^	0.85	<0.050
Week 5, %						
Injurious interaction	0.1	0.2	0.2	0.1	0.06	0.537
Positive interaction	1.7	2.3	3.0	2.0	0.44	0.376
Exploration	2.5 ^c^	6.2 ^a^	4.8 ^ab^	3.7 ^bc^	0.31	<0.001
Eating/drinking	4.8	5.5	5.4	4.9	0.39	0.600
Inactivity	90.6 ^a^	85.6 ^b^	86.3 ^b^	89.0 ^ab^	0.62	<0.050
Week 8, %						
Injurious interaction	0.2	0.3	0.2	0.1	0.07	0.375
Positive interaction	1.1	1.4	1.4	0.9	0.25	0.561
Exploration	2.7	4.7	4.4	3.7	0.64	0.076
Eating/drinking	5.0	5.1	5.2	4.1	0.44	0.051
Inactivity	90.7 ^ab^	88.3 ^c^	88.7 ^bc^	90.9 ^a^	1.13	<0.050
Week 11, %						
Injurious interaction	0.8	0.2	0.3	0.6	0.18	0.290
Positive interaction	0.3	0.5	0.5	0.5	0.08	0.444
Exploration	3.9 ^b^	5.0 ^ab^	5.8 ^a^	5.0 ^ab^	0.45	<0.050
Eating/drinking	5.4	4.1	4.5	4.5	0.71	0.691
Inactivity	89.3	90.0	88.6	89.1	1.18	0.875

^a, b, c^ Within a row, values with different superscript letters differ (*p* < 0.05). Data are presented as mean  ±  SEM. EMs, enrichment materials; RS, rice-straw silage; SD, sawdust; SB, sling belt; SEM, standard error of the mean. *n* refers to the total number of animals assessed. The statistical model included ‘Pen’ as a random effect, ensuring that the pen served as the valid experimental unit.

**Table 5:** Effects of the provision of EMs on the proportion of body lesions in fattening pigs are displayed by treatment and experimental week.

Item	Control	RS	SD	SB	SEM	*p* Value
	*n* = 88	*n* = 84	*n* = 84	*n* = 88		
Week 0, %						
Score 0	69.9	41.6	55.9	73.9	7.32	0.115
Score 1	28.8	46.4	35.7	21.7	7.05	0.243
Score 2	1.2	11.9	8.3	4.3	4.21	0.320
Week 3, %						
Score 0	60.7	48.2	50.9	67.0	4.17	0.074
Score 1	36.4	44.6	38.8	27.9	3.90	0.154
Score 2	2.8	7.1	10.1	5.1	3.25	0.459
Week 6, %						
Score 0	54.2	67.7	56.8	65.2	5.08	0.363
Score 1	39.4	30.5	35.8	29.5	4.20	0.468
Score 2	6.3	1.8	7.2	5.2	2.12	0.412
Week 10, %						
Score 0	56.0 ^b^	82.9 ^a^	75.9 ^ab^	68.0 ^ab^	3.72	0.021
Score 1	37.0 ^a^	16.4 ^b^	22.8 ^ab^	28.5 ^ab^	2.84	0.020
Score 2	7.0 ^a^	0.6 ^b^	1.2 ^ab^	3.5 ^ab^	1.27	0.044
Weeks 0–10, %						
Score 0	58.4	65.4	63.2	67.5	1.91	0.074
Score 1	36.7 ^a^	30.7 ^ab^	31.0 ^ab^	28.2 ^b^	1.47	0.038
Score 2	4.9	3.9	5.7	2.2	1.71	0.457

^a, b^ Within a row, values with different superscripts letters differ (*p* < 0.05). Data are presented as the mean  ±  SEM. EMs, enrichment materials; RS, rice-straw silage; SD, sawdust; SB, sling belt; SEM, standard error of the mean. *n* refers to the total number of animals assessed. The statistical model included ‘Pen’ as a random effect, ensuring that the pen served as the valid experimental unit.

**Table 6:** Effects of the provision of EMs on environmental conditions in pens at morning (09:00) and afternoon (15:00) in week 10 are displayed by treatment.

Item	Control	RS	SD	SB	SEM	*p* Value
At 09:00 (Week 10)						
NH_3_, mg/m^3^	43.9	55.1	62.5	38.0	6.11	0.074
CO_2_, mg/m^3^	6751.2	8092.1	7463.4	7017.0	436.26	0.210
Relative humidity, %	77.8	81.5	82.1	77.3	2.38	0.503
Temperature, °C	25.6	24.2	25.5	25.4	0.78	0.338
PM_2.5_, mg/m^3^	36.7	80.6	40.5	47.6	21.10	0.292
PM_10_, mg/m^3^	101.9	174.6	98.5	133.5	31.46	0.258
At 15:00 (Week 10)						
NH_3_, mg/m^3^	32.8 ^bc^	48.2 ^ab^	52.3 ^a^	29.2 ^c^	5.69	<0.001
CO_2_, mg/m^3^	4126.4 ^c^	5315.1 ^ab^	5454.2 ^ab^	4588.5 ^bc^	470.50	<0.05
Relative humidity, %	62.5	67.7	68.9	62.9	1.75	0.073
Temperature, °C	27.0	27.2	27.3	26.7	0.48	0.422
PM_2.5_, mg/m^3^	19.3	16.8	15.5	20.0	1.17	0.084
PM_10_, mg/m^3^	126.7 ^a^	95.5 ^b^	79.5 ^b^	128.4 ^a^	12.56	0.044

^a, b, c^ Within a row, values with different superscripts letters differ (*p* < 0.05). Data are presented as the mean  ±  SEM. EMs, enrichment materials; PM, particulate matter; RS, rice-straw silage; SD, sawdust; SB, sling belt; SEM, standard error of the mean. Treatment was included as a fixed effect and pen (treatment) as a random effect; pen was considered the experimental unit, with three replicate pens per treatment.

## Data Availability

The data presented in this study are available upon request from the corresponding author.

## Supplementary Materials

The following supporting information can be downloaded at: https://www.mdpi.com/article/10.3390/ani16040580/s1. Supplementary Table S1. Effects of provision of EMs on proportion of ear lesions in fattening pigs are displayed by treatment and experimental week.

## Abbreviations

The following abbreviations are used in this manuscript:

ADG	Average daily gain
ADFI	Average daily feed intake
EMs	Enrichment materials
G:F	Gain-to-Feed ratio
PM	Particulate matter
RS	Rice-straw silage
SEM	Standard errors of the mean
SD	Sawdust
SB	Sling belt
Wk	Week
