# Supplementary material for: Effects of Housing and Environmental Enrichment on Performance, Welfare, and Air Quality in Fattening Pigs
Source: Animals (Basel). 2026 Feb 12;16(4):580. doi: 10.3390/ani16040580 (PMC12937388; doi:10.3390/ani16040580)
Supplement: Supplementary file 1 [file animals-16-00580-s001.zip › animals-4081861-supplementary.pdf]

animals

MDPI

Supplementary Table

Supplementary Table S1. Effects of provision of EMs on proportion of ear lesions in fattening pigs are displayed by treatment and experimental week.

Item	Control	RS	SD	SB	SEM	*p* value
	n = 88	*n* = 84	*n* = 84	*n* = 88		
Week 0, %						
Score 0	100.0	100.0	100.0	100.0	0.00	Non-est
Score 1	0.0	0.0	0.0	0.0	0.00	Non-est
Score 2	0.0	0.0	0.0	0.0	0.00	Non-est
Week 3, %						
Score 0	98.4	99.4	97.0	98.2	1.78	0.714
Score 1	0.5	0.0	1.8	0.6	0.98	0.626
Score 2	1.0	0.6	1.2	1.1	0.89	0.837
Week 6, %						
Score 0	92.9	98.8	94.6	96.6	3.04	0.431
Score 1	5.4	0.6	4.1	2.3	2.32	0.415
Score 2	1.6	0.6	1.2	1.1	0.85	0.679
Week 10, %						
Score 0	86.8	96.9	91.4	93.6	3.91	0.290
Score 1	12.5	3.0	8.5	6.3	4.09	0.362
Score 2	0.6	0.0	0.0	0.0	0.31	0.441
Weeks 0–10, %						
Score 0	92.6	98.2	94.6	95.7	2.71	0.437
Score 1	6.5	1.5	4.8	3.7	2.42	0.451
Score 2	0.8	0.3	0.6	0.5	0.43	0.661

Data are presented as the mean ± SEM. EMs, enrichment materials; RS, Rice-straw silage; SD, Sawdust; SB, Sling belt; SEM, standard error of the mean. *n* refers to the total number of animals assessed. The statistical model included 'Pen' as a random effect, ensuring that the pen served as the valid experimental unit.
